# Linking Intermediate to Final “Real-World” Outcomes: Is Financial Toxicity a Reliable Predictor of Poorer Outcomes in Cancer?

**DOI:** 10.3390/curroncol29040202

**Published:** 2022-04-02

**Authors:** Christopher J. Longo

**Affiliations:** 1 Health Policy and Management, DeGroote School of Business, McMaster University, Burlington, ON L7L 5R8, Canada; cjlongo@mcmaster.ca; 2 Dalla Lana School of Public Health, University of Toronto, Toronto, ON L8S 4L8, Canada

**Keywords:** cost-effectiveness, financial toxicity, quality of life, overall survival, foregone care

## Abstract

Traditionally, economic evaluations are based on clinical trials with well-defined patient populations that exclude many patient types. By contrast, studies that incorporate general patient populations end up including those in lower income categories, some of whom have significant financial burdens (often described as financial toxicity) related to their care. Consideration of these patient burdens when examining the incremental cost-effectiveness of newer treatments from a clinical trial perspective can result in differing conclusions regarding cost-effectiveness. The challenge is to reliably assess the link between financial toxicity, quality of life and potential decisions to forego or delay care. It is also well-documented that these financial effects are not evenly distributed across populations, with those with low income and of black or Latino decent being most affected. There is a paucity of literature in this space, but some of the early work has suggested that for lung, breast, colorectal and ovarian cancers there are poorer quality-of-life scores and/or shorter overall survival for those experiencing financial toxicity. Hence, we may see both a lower quality of life and a shorter duration of life for these populations. If this is the case, additional considerations include: are the benefits of newer, more-expensive treatment strategies muted by the lack of adherence to these newer treatments due to financial concerns, and, if true, can these effects be effectively quantified as “real-world” outcomes? This rapid review examines these possibilities and the steps that may be required to examine this reliably.

## 1. Introduction

Traditionally, economic evaluations are based on clinical trials using well-defined populations that exclude many patient types. By contrast, “real-world” studies that incorporate general patient populations include those in lower income categories, some of whom have significant financial burdens related to their care. These financial burdens, in extreme cases, are often described as financial toxicity. Financial toxicity has been defined as the “distress and hardship arising from the financial burden of cancer treatment” [1]. This financial toxicity can be measured objectively [2] or subjectively [3] using existing measurement tools, with differences evident across countries and health systems.

The consideration of these patient burdens when examining the incremental cost-effectiveness of newer treatments from the clinical trial perspective can result in different conclusions regarding their cost-effectiveness. More difficult to assess is the reliability of this link between financial toxicity, quality of life and potential decisions to forego or delay care. It is also well documented that these financial effects are not evenly distributed across populations, with those with low income and of black or Latino decent being most affected [4].

There are two possible reasons for the negative impact on incremental cost-effectiveness ratios (ICER), and both impact the denominator in the ICER. First, high financial burden due to out-of-pocket costs (OOPC) has been shown to decrease quality of life. These OOPC typically include copayments or costs associated with medical care at health facilities or at home and can include cost of travel to and parking at these facilities. Second, these same financial burdens often result in decisions to forego or delay care, which, in turn can result in greater morbidity and mortality. In the elderly, these kinds of delays or foregoing of treatment for oral chemotherapy are not uncommon, with rates of delay/discontinuation reaching 70% for the most expensive oral agents [5]. In 2009, the impact related to high out-of-pocket costs for medications suggested that the newer oral immunotherapies could have copayments higher than USD 500 per claim for 9% of the study sample [6]. These costs have continued to rise for cancer treatments and due to the increasing use of orally administered therapies [7]. Yezefski [7] suggests that by 2025 these out-of-pocket costs could reach $57,000 annually for Medicare patients in the US. These high copayments are likely to result in even more decisions to forego or delay care. The focus in this examination is with developed countries, as developing countries often do not measure utility directly, but rather use disability-adjusted-life-years that assign values based on illness.

Do these decisions to forego/delay care consistently result in poorer clinical outcomes for patients? If the answer is “yes” to this first question, then the cost-effectiveness of the strategy for these populations generally becomes less attractive because of the poorer outcomes.

There is a paucity of literature in this space, but our rapid review suggests some of the early work has shown that lung, breast, colorectal and ovarian cancers result in poorer quality-of-life scores and/or shorter overall survival for those with financial toxicity. It is also well documented that these effects are not evenly distributed across populations, with those with low income and black or Latino decent being most affected [4]. If this is the case, we need to investigate whether the benefits of newer, more-expensive treatment strategies are muted by the lack of adherence to these newer treatments due to financial concerns; if true, we can then ask whether these effects can be accurately quantified. This review examines these possibilities and the steps that may be required to examine this phenomenon reliably.

## 2. Search Methods

**Figure 1:**
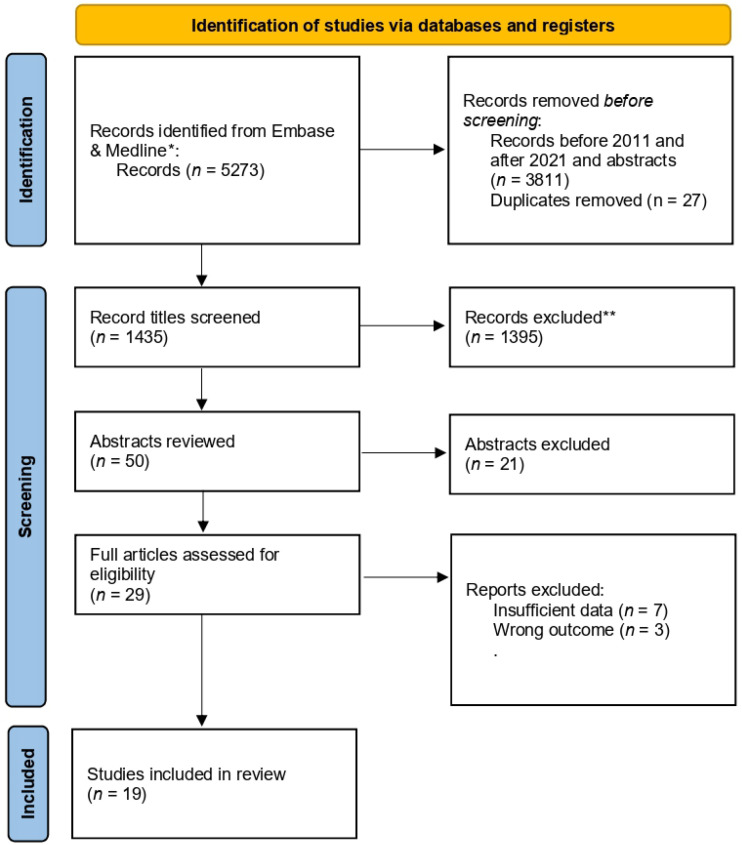
PRISMA flow diagram “Financial toxicity, quality of life and survival” (dated 25 March 2022). * Consider, if feasible to do so, reporting the number of records identified from each database or register searched (rather than the total number across all databases/registers). ** If automation tools were used, indicate how many records were excluded by a human and how many were excluded by automation tools.

A search of Medline (1996 to present) and Embase (1996 to present) was undertaken with the search terms “out-of-pocket *” or “financial *” (includes: toxicity, distress, stress, strain, problems) or “patient burden” or “patient cost” or “foregone care” and “cancer” or “oncol *” with abstracts excluded and with date restrictions of 1 January 2011 to 31 December 2021, plus a hand-search was conducted for selected articles (Figure 1).

## 3. Current Evidence

As mentioned, based on the rapid review, the published work in this space appears to be limited. The following paragraphs examine literature related to three impacts of financial toxicity: reduced quality of life, reduced overall survival and risks based on patient characteristics.

### 3.1. Reduced Quality of Life

Literature has shown that lung cancer patients with financial toxicity have a lower quality of life than those who do not have financial toxicity (correlation coefficient, 0.41; *p* < 0.001) related to lung cancer treatments [8]. Additionally, an Italian study on breast, lung and ovarian cancers found that financial burden was predictive of a worse baseline quality-of-life response (OR 1.35; 95% CI 1.09–1.70; *p* = 0.009) [9]. Delgado-Guay [10] found that financial distress in cancer patients correlated with statistically significant decreases in the FACT-G quality-of-life score (Spearman correlation; r = −0.23, *p* = 0.0057). Fenn [11] found a four-fold decrease in reporting a quality of life of “good” or “better” between those with financial toxicity versus those who reported “no financial problems” (OR 0.24; 95% CI 0.14 to 0.40). Lathan [12] found that colorectal and lung patient populations with lower financial reserves (2 months or less) had a lower quality-of-life score (EQ-5D) compared to those who had 12 months of financial reserves (4.70 vs. 5.22). Examination of the relationship between financial burden and quality of life using structural equation modeling showed poorer quality of life for those experiencing a significant financial burden (adjusted beta −0.06 per burden category on a five-point burden scale) even after adjusting for the effects of income, employment, disease status, comorbidities and other potential contributors of health-related quality of life [13].

### 3.2. Reduced Overall Survival

It has been shown that poor coverage (less chemotherapy) translates to poorer overall care in breast, colorectal, lung and ovarian cancers for Medicare or dual-eligible beneficiaries—although no specific statement on outcomes was made, not receiving treatment would suggest poorer survival [14]. Studies have shown that financial toxicity that results in bankruptcy increases the risk of mortality, including one specific study that found the hazard ratio was 1.79 (95% CI 1.64 to 1.96), with even higher ratios for colorectal, prostate and thyroid cancers [15]. Ma et al. showed that those with high financial toxicity exhibited worse overall survival (HR 1.75, 95% CI 1.05–2.94, *p* = 0.03) and cancer-specific survival (HR 2.28, 95% CI 1.31–3.96, *p* = 0.003) [16]. Klein et al. showed that increasing pre-treatment financial toxicity was associated with shorter progression-free survival (*p* = 0.011) [17]. An Italian study examined a follow-up-from-baseline questionnaire completed during treatment showing that those who developed financial toxicity (22.5%) had an increased risk of death (HR 1.20; 95% CI 1.05–1.37; *p* = 0.007) [9].

### 3.3. Risks Based on Patient Characteristics

At a country level, countries with more than 20% of the population living below the poverty line have on average a 13% higher death rate for men and a 3% higher rate for women when compared to countries with poverty rates lower than 10% [18]. Warren [14] has noted that the level of coverage in the US impacts the likelihood of access to chemotherapy. Even in the case of dual-eligibility (Medicare and Medicaid) those on Medicare with private insurance were more likely to receive chemotherapy (44.2% vs. 60.8%). Additionally, work by Kent et al. [19] suggests that those who are under financial pressures are more likely to forego (13.5% vs. 5.1%; *p* < 0.0001) or delay care (18.1% vs. 7.4%; *p* < 0.0001) when compared to those without financial pressures, and that this is particularly problematic for younger populations and those of a minority race/ethnicity.

Interestingly, there are a number of papers published that propose interventions to try to mitigate some of these patient and system factors, particularly for those populations known to be at higher risk. The literature suggests that the harm is not evenly distributed across populations, with the elderly at elevated risk [20]. Additionally, introducing higher-quality colorectal cancer care to the vulnerable has demonstrated improved overall survival in this population [21]. Several local, regional, and national programs in the US have been initiated since 2000 to mitigate the disparities related to screening and diagnostics for early detection of common cancers for underserved populations, with encouraging results [18].

### 3.4. Implications

The data presented here suggest that financial toxicity can negatively impact quality of life and overall survival and is known to be more prevalent in those with low income, of minority race/ethnicity, the young and the elderly. As stated, cost-effectiveness based on clinical trials will miss these effects, as typically many of these extra costs are borne by the pharmaceutical companies. In this regard, they miss most of the negative effects of costs related to copayments or coverage limits, which end up being borne by patients. This also means decisions to forego or delay care for financial reasons are understated or absent in these trials. Our rapid review suggests that this financial toxicity is likely to affect both quality of life and length of life; when examining a cost-effectiveness ratio, these reductions in life-years and quality-adjusted-life-years will increase the ICER, making it less attractive. In essence, many clinical trial results will improve the cost-effectiveness ratio by excluding these known effects.

## 4. Future Research

The data shared in this manuscript provide early signals of the potential negative effects of financial toxicity on overall QoL and survival in cancer patients.

What is needed is a more systematic way of evaluating these negative effects on both quality of life and reductions in overall survival, including the incidence rates of financial toxicity in the general cancer population. It is likely that these effects will vary by tumor type, stage of disease and even by the currently available treatment options in each country or jurisdiction. Ideally, this research will be an ongoing examination, with updates on a regular basis to see how increased pricing for cancer treatments (both pharmaceutical and non-pharmaceutical) impact quality of life and overall survival for cancer patients.

Economic modeling might be the best possible way forward, using anonymized estimates based on income and family assets generally available through government agencies. However, at least some of this data must come from prospective data, such as the articles highlighted in this manuscript, to help inform the modeling inputs. These two sources will help generate dis-utilities related to QoL and hazard ratios related to overall survival associated with financial toxicity that could be added to existing industry and government cancer models currently in use in a variety of countries.

Other papers I reviewed offer suggestions on how we might mitigate the financial toxicity and, by doing so, reduce the negative effects on outcomes [14,20]. Partly, this requires understanding the barriers to care, particularly those barriers that are linked to income or socioeconomic status. Ideally, introduction of these factors could also be included as a model input.

These findings are important both to governments and the pharmaceutical industry. To date, these effects are not captured in most economic evaluations (nor are the strategies to mitigate these effects). This early work provides some food for thought on why those in the lower income quartiles are more likely to have poorer outcomes, as well as offering suggestions on how to mitigate these effects.

It is hoped that additional research in this area over the coming years will shed more light on these effects and possibly introduce a better way forward for the benefit of those populations at greatest risk.

## 5. Discussion

This short manuscript has highlighted some interesting research on the link between financial toxicity and poorer quality of life and survival outcomes, and likely more accurately reflects “real-world” experience.

This manuscript is, at present, hypothetical in nature, and a number of limitations of this potential new way forward remain. Firstly, the data we have today are not necessarily representative study samples and are often smaller studies. Hopefully, future work will continue and ideally be organized on a larger scale that is more population-based. Secondly, modeling reliability is directly related to the quality of the evidence—current data might be sufficient for certain cancers, but many gaps likely exist across the cancer spectrum. It is hoped that, going forward, as more primary data is collected, a more comprehensive picture of financial toxicity across all cancers will be available in the not-too-distant future. Thirdly, the link between financial toxicity and reductions in QoL or death is dependent on a number of other factors (income, education and ethnic origin) and, ideally, researchers should try to separate these factors to see if effect size is different in each of these groups. As with the other limitations discussed, more primary data collection is needed to adjust for these factors. Lastly, does the link between copayments and outcomes translate into poorer adherence, which may be driving the QoL and survival outcomes? Again, more primary data is needed to better answer this question. 

## 6. Conclusions

Our review suggests that financial toxicity can have a negative impact on quality of life and overall survival. It is hoped that this limited data, along with a willingness to continue to collect this data, may lead to the development of modified industry and government economic models that will take these financial factors into account; these could even be specific to a jurisdiction or by cancer type. Concurrently, we could look to finding ways to eliminate or mitigate some of the financial burdens for the most vulnerable, as has been shown in some of the highlighted literature. An opportunity for policy makers also exists to provide incentives—or, better yet, requirements—that ensure these factors be required for health economic analyses provided by governments and industry alike.
